# Distinct Migratory Properties of M1, M2, and Resident Macrophages Are Regulated by α_D_β_2_ and α_M_β_2_ Integrin-Mediated Adhesion

**DOI:** 10.3389/fimmu.2018.02650

**Published:** 2018-11-15

**Authors:** Kui Cui, Christopher L. Ardell, Nataly P. Podolnikova, Valentin P. Yakubenko

**Affiliations:** ^1^Department of Biomedical Sciences, Center of Excellence for Inflammation, Infectious Disease and Immunity, Quillen College of Medicine, East Tennessee State University, Johnson City, TN, United States; ^2^Center for Metabolic and Vascular Biology, School of Life Sciences, Arizona State University, Tempe, AZ, United States

**Keywords:** integrin α_D_β_2_(CD11d/CD18), integrin α_M_β_2_(CD11b/CD18), macrophages (M1/M2), migration, inflammation, adhesive receptors

## Abstract

Chronic inflammation is essential mechanism during the development of cardiovascular and metabolic diseases. The outcome of diseases depends on the balance between the migration/accumulation of pro-inflammatory (M1) and anti-inflammatory (M2) macrophages in damaged tissue. The mechanism of macrophage migration and subsequent accumulation is still not fully understood. Currently, the amoeboid adhesion-independent motility is considered essential for leukocyte migration in the three-dimensional environment. We challenge this hypothesis by studying the contribution of leukocyte adhesive receptors, integrins α_M_β_2_, and α_D_β_2_, to three-dimensional migration of M1-polarized, M2-polarized, and resident macrophages. Both integrins have a moderate expression on M2 macrophages, while α_D_β_2_ is upregulated on M1 and α_M_β_2_ demonstrates high expression on resident macrophages. The level of integrin expression determines its contribution to macrophage migration. Namely, intermediate expression supports macrophage migration, while a high integrin density inhibits it. Using *in vitro* three-dimensional migration and *in vivo* tracking of adoptively-transferred fluorescently-labeled macrophages during the resolution of inflammation, we found that strong adhesion of M1-activated macrophages translates to weak 3D migration, while moderate adhesion of M2-activated macrophages generates dynamic motility. Reduced migration of M1 macrophages depends on the high expression of α_D_β_2_, since α_D_-deficiency decreased M1 macrophage adhesion and improved migration in fibrin matrix and peritoneal tissue. Similarly, the high expression of α_M_β_2_ on resident macrophages prevents their amoeboid migration, which is markedly increased in α_M_-deficient macrophages. In contrast, α_D_- and α_M_-knockouts decrease the migration of M2 macrophages, demonstrating that moderate integrin expression supports cell motility. The results were confirmed in a diet-induced diabetes model. α_D_ deficiency prevents the retention of inflammatory macrophages in adipose tissue and improves metabolic parameters, while α_M_ deficiency does not affect macrophage accumulation. Summarizing, β_2_ integrin-mediated adhesion may inhibit amoeboid and mesenchymal macrophage migration or support mesenchymal migration in tissue, and, therefore, represents an important target to control inflammation.

## Introduction

Monocyte/macrophage migration to, and accumulation within the site of inflammation are critical steps in the development of the inflammatory response. While acute inflammation is usually generated as a defensive mechanism, the development of chronic inflammation is an essential step in the initiation or progression of many devastating diseases including atherosclerosis, diabetes, obesity, arthritis and others (1–4). Macrophage accumulation at the damaged tissue is a hallmark of inflammation (5, 6). However, the particular subset of accumulated macrophages is critical for the further development or resolution of chronic inflammation. Classically activated (M1) macrophages produce a harsh pro-inflammatory response, while alternatively activated (M2) macrophages may have anti-inflammatory functions (7, 8). The balance between the accumulation of pro-inflammatory and anti-inflammatory macrophages regulates the fate of inflammation. So far, the mechanism of macrophage accumulation is not fully understood.

Macrophage accumulation at the site of inflammation depends upon monocyte recruitment, macrophage retention and emigration. Monocyte recruitment includes activation, diapedesis through the endothelial monolayer (2D migration) (9, 10), and migration through the extracellular matrix to the site of inflammation (3D migration). While the role of leukocyte adhesive receptors in 2D migration is well-established (9, 11), their contribution to macrophage migration through 3D extracellular matrix (ECM) is still unclear. Macrophages utilize two types of motility in a 3D environment—amoeboid and mesenchymal. Amoeboid migration is adhesion-independent movement that is based on flowing and squeezing. This migratory mode was shown to be dominant for neutrophils, dendritic cells and lymphocytes (12). Mesenchymal migration involves the classical adhesion-mediated mechanism that includes cell protrusion and adhesion of the leading edge, followed by detachment of the trailing edge and retraction of the contractile cell rear (13). It has been shown that cell-substratum adhesiveness regulates the fate of mesenchymal cell migration. Namely, an intermediate level of adhesiveness generates the optimal conditions for cell migration (14). Low adhesiveness does not support cell motility, while a very high level of adhesiveness thwarts cell locomotion because it inhibits cell detachment from the substrate (15, 16). The density of adhesive receptors on the cell surface is one of the most critical parameters of cell-substratum adhesiveness. Therefore, a high density of cell adhesion receptors that generate a high adhesiveness may lead to the retention of cells (15, 17).

Integrins are the most important cell adhesive receptors that are involved in monocyte/macrophage migration. Of particular note is the subfamily of β_2_ integrins that are exclusively expressed on leukocytes and consist of four members: α_L_β_2_ (CD11a/CD18), α_M_β_2_ (CD11b/CD18), α_X_β_2_ (CD11c/CD18), and α_D_β_2_ (CD11d/CD18) (18). Integrins α_M_β_2_ and α_D_β_2_ are the most interesting members with regard to cell migration, since α_L_β_2_ has no ligands in ECM (19) and α_X_β_2_ demonstrated a very low expression on macrophages (20). In contrast, α_M_ and α_D_ have marked macrophage expression and share many ECM ligands (21, 22).

Different subsets of macrophages have a diverse expression of integrins (23) and, most importantly, possess different migratory characteristics (24). We hypothesize that integrin expression regulates the distinct migratory properties of M1-polarized, M2-polarized, and resident macrophages. We realize that *in vitro* activated M1 and M2 macrophages do not fully represent the varieties of pro-inflammatory and anti-inflammatory macrophages *in vivo*; however, these cells are appropriate models that can help us to understand the migratory mechanisms of different macrophage subsets during inflammatory diseases.

In our previous project, we found that the pro-atherogenic role of integrin α_D_β_2_ depends upon the upregulation of α_D_ on pro-inflammatory M1 macrophages *in vitro* and on macrophages in atherosclerotic lesions, which apparently mediates macrophage retention (23). In agreement with this, α_D_-deficiency reduced the development of atherosclerosis and released the migration of M1 macrophages *in vitro* (23).

In this paper we further develop this project by analysing the role of β_2_ integrins on different subsets of macrophages and attempt to depict the mechanisms that stimulate cell migration/retention based on the analysis of integrin expression, cell adhesion, secretion of proteases, and mode of cell migration. We found a strong correlation between macrophage migration and expression of α_M_β_2_ and α_D_β_2_. A moderate expression of α_M_β_2_ and α_D_β_2_ on M2 macrophages supports cell movement, while the upregulation of α_D_β_2_ on M1 macrophages and α_M_ on resident macrophages prevents mesenchymal and/or amoeboid migration. These results were verified by using α_M_- and α_D_-deficient macrophages in 3D *in vitro* migration and by using an *in vivo* model for the resolution of peritoneal inflammation and diet-induced diabetes.

Therefore, the regulation of β_2_ integrin expression may help to shift the pro-/anti- inflammatory balance at the site of inflammation and reduced the pathophysiological outcome.

## Materials and methods

### Reagents and antibodies

Reagents were purchased from Sigma-Aldrich (St. Louis, MO, United States) and Thermo Fisher Scientific (Waltham, MA, United States). Rock inhibitor (Y27632) and aprotinin were from Sigma-Aldrich. Recombinant human and mouse IFNγ, IL-4, MCP-1, and FMLP were purchased from Invitrogen Corporation (Carlsbad, CA, United States). Anti-human α_D_ mAb (clone 240I) was generously provided by Eli Lilly Corporation (Indianapolis, IN, United States). Polyclonal antibody against the α_D_ I-domain was described previously (10). The antibody recognizes both human and mouse α_D_ I-domains and has no cross-reactivity with recombinant human and mouse α_M_, α_X_, and α_L_ I-domains. The antibody was isolated from rabbit serum by affinity chromatography using α_D_I-domain-Sepharose. Mouse PE-cy7 and APC- conjugated anti-α_M_ mAb (clone M1/70) and F4/80 mAbs were from eBioscience (San Diego, CA, United States). The mAb 44a directed against the human α_M_ integrin subunit was purified from the conditioned media of the hybridoma cell line obtained from American Type Culture Collection (ATCC, Manassas, VA, United States) using protein A agarose (GE Healthcare, Piscataway, NJ, United States).

### Animals

Wild type (C57BL/6J, stock # 000664) and integrin α_D_-deficient (B6.129S7-*Itgad*^*tm1Bll*^/J, stock # 005258 and integrin α_M_-deficient (B6.129S4-*Itgam*^*tm1Myd*^/J, stock # 003991) mice were bought from Jackson Laboratory (Bar Harbor, ME). α_D_-deficient and α_M_-deficient mice have been backcrossed to C57BL/6 for at least ten generations. All procedures were performed according to animal protocols approved by East Tennessee State University IACUC.

### Flow cytometry analysis

Flow cytometry analysis was performed to assess the expression of α_D_ and α_M_ on mouse peritoneal macrophages. Cells were harvested and pre-incubated with 4% normal goat serum for 30 min at 4°C, then 2 × 10^6^ cells were incubated with specific antibody for 30 min at 4°C. Non-conjugated antibodies required additional incubation with Alexa 488 or PE-cy7-donkey anti-mouse IgG (at a 1:1,000 dilution) for 30 min at 4°C. Finally, the cells were washed and analyzed using a Fortessa X-20 (Becton Dickinson).

### Generation of classically activated (M1) and alternatively activated (M2) mouse macrophages

Peritoneal macrophages from 8 to 12 week old mice (WT and $\alpha_{\text{D}}^{-/-}$, *n* = 3 mice per group) were harvested by lavage of the peritoneal cavity with 5 ml of sterile PBS 3 days after intraperitoneal (IP) injection of 4% thioglycollate (TG; 0.5 ml). The cells were washed twice with PBS and resuspended in complete RPMI media. The cell suspension was transferred into 100 mm petri dishes and incubated for 2 h at 37°C in humidified air containing 5% CO_2_ atmosphere. Non-adherent cells were washed out with RPMI media, and the adherent macrophages were replenished with RPMI media. The macrophages were differentiated to M1 and M2 phenotypes by treatment with recombinant mouse interferon-γ (IFN-γ) (100 U/ml, Thermo Fisher) and interleukin 4 (IL-4) (2 nM, Thermo Fisher), respectively, for 4 days. Medium with IFN-γ and IL-4 were changed every 2 days or as required. The M1 phenotype macrophages from WT and $\alpha_{\text{D}}^{-/-}$ were labeled with red fluorescent marker PKH26 and green fluorescent marker PKH67, respectively, according to the manufacturer's instructions (Sigma-Aldrich). The fluorescently-labeled cells were dissociated from the plates using 5 mM EDTA in PBS and used for the experiments thereafter.

### Cell adhesion assay

The adhesion assay was performed as described previously (22) with modifications. Briefly, 96-well plates (Immulon 2HB, Cambridge, MA, United States) were coated with different concentrations of fibrinogen or Matrigel for 3 h at 37°C. The wells were post-coated with 0.5% polyvinyl alcohol for 1 h at 37°C. Mouse peritoneal macrophages or HEK 293 cells transfected with α_M_β_2_, or α_D_β_2_ integrins were labeled with 10 μM Calcein AM (Molecular Probes, Eugene, OR) for 30 min at 37°C and washed with DMEM and resuspended in the same medium at a concentration of 1 × 10^6^ cells/mL. Aliquots (50 μL) of the labeled cells were added to each well. For inhibition experiments, cells were mixed with antibodies and incubated for 15 min at 22°C before they were added to the coated wells. After 30 min of incubation at 37°C in a 5% CO_2_ humidified atmosphere, the non-adherent cells were removed by washing with HBSS. The fluorescence was measured in a Synergy H1 fluorescence plate reader (BioTek, Winooski, VT, United States), and the number of adherent cells was determined from a labeled control.

### Migration of macrophages in 3D fibrin gel and matrigel

The migration assay was performed as described previously (25). WT and $\alpha_{\text{D}}^{-/-}$ or WT and $\alpha_{\text{M}}^{-/-}$ peritoneal macrophages activated to M1 or M2 phenotype as described above were labeled with PKH26 red fluorescent dye and PKH67 green fluorescent dye, respectively. Cell migration assay was performed for 48 h at 37°C in 5% CO_2_ in a sterile condition. An equal number of WT and $\alpha_{\text{D}}^{-/-}$ macrophages was evaluated by cytospin of mixed cells before the experiment and at the starting point before migration. Labeled WT (1.5 × 10^5^) and $\alpha_{\text{D}}^{-/-}$ (1.5 × 10^5^) activated macrophages were plated on the membranes of transwell inserts with a pore size of 8 μm and 6.5 mm in diameter (Costar, Corning, NY) precoated with fibrinogen (Fg). Fibrin gel (100 μl/sample) was made by 0.75 mg/ml Fg containing 1% FBS and 1% P/S and activated by 0.5 U/ml thrombin. Matrigel (50%) was diluted by RPMI-1640 supplemented with 1% FBS and 1% P/S. 30 nM of MCP-1 (or 100 nM FMLP) were added on the top of the gel to initiate the migration. Migrating cells were detected by Leica Confocal microscope (Leica-TCS SP8) and the results were analyzed and reconstructed using IMARIS 8.0 software.

### Adoptive transfer in the model of resolution of peritoneal inflammation

Adoptive transfer was performed as described previously (23). Briefly, fluorescently-labeled WT (red PKH26 dye) and $\alpha_{\text{D}}^{-/-}$ or $\alpha_{\text{M}}^{-/-}$ (green PKH67 dye) M1- or M2-activated macrophages were mixed in a 1:1 ratio and further injected intraperitoneally into wildtype mice at 4 days after thioglycollate (TG)-induced inflammation. 3 days later, peritoneal macrophages were harvested with 5 ml PBS supplemented with 5 mM EDTA. The percentages of red and green fluorescent macrophages in the peritoneal exudate were assessed by fluorescence microscopy, multi-color flow cytometer (Fortessa X-20) and imaging flow cytometry (ImageStream Mark II, Amnis).

The PKH26 and PKH67 dyes were switched in one experiment to verify the effect of dye on cell migration. We did not detect any difference between two dyes. The quantification of the data was analyzed by using Image Analysis Software (EVOS, Thermo Fisher).

### Adoptive transfer in the model of diet-induced diabetes

The approach is based on previously published method (26) with some modifications. Monocytes were isolated from the bone marrow progenitors of WT and α_D_-deficient mice using magnetic bead separation kit (Miltenyi Biotec, Gaithersburg, MD, United States). Monocytes were labeled with red, PKH26 (WT) or green, PKH67 ($\alpha_{\text{D}}^{-/-}$) fluorescent dyes. Red (1.5 × 10^6^) and green (1.5 × 10^6^) cells were mixed together and injected in tail vein of wild type C57BL6 mice fed high fat diet (45% kcal/fat) for 8 weeks. After 3 days adipose tissue was isolated, digested as described previously (26) and analyzed using FACS (Fortessa X-20, BD, United States) and imaging flow cytometry (ImageStream Mark II, Amnis).

### Glucose tolerance and insulin sensitivity tests

Wild type and $\alpha_{\text{D}}^{-/-}$ mice fed a high fat diet for 16 weeks were fasted overnight in a new cage containing water but no food, (~16 h). The following morning mice were weighed, and an initial blood glucose level was measured using a glucometer and blood from the tail vein. Glucose (2 grams/kg body weight of 20% D-glucose) was administered IP and at 15, 30, 60, and 120 min post injection blood glucose was again measured.

For insulin sensitivity test, mice fed a high fat diet were fasted for 5 h, starting at 7 a.m. (lights on). After fasting, mice were weighed, and the initial level of blood glucose measured as described above. Insulin (0.75 mU/g) was injected I.P. and the level of blood glucose was evaluated at 15, 30, 45, and 60 min.

### Quantitative RT-PCR

Cellular mRNA was extracted from macrophages using the Qiagen Oligotex mRNA Midi Kit. mRNA was reverse transcribed with the iScript cDNA Synthesis Kit (Bio-Rad Laboratories, Inc., Hercules, CA, United States) and real-time quantitative PCR was performed using SYBR Green Supermix (Bio-Rad) on an MyIQ2 two color real-time PCR detection system (Bio-Rad), with the thermal cycler conditions suggested by the manufacturer. The sequences of integrin primers are shown below: α_D_ forward, 5′-GGAACCGAATCAAGGTCAAGTA-3′, and reverse, 5′-ATCCATTGAGAGAGCTGAGCTG-3′. α_M_ forward, 5′-TCCGGTAGCATCAACAACAT-3′ and reverse, 5′-GGTGAAGTGAATCCGGAACT-3′. α_4_ forward, 5′-AAGGAAGCCAGCGTTCATATT-3′, and reverse, 5′-TCATCATTGCTTTTGCTGTTG-3′. α_5_ forward, 5′-CAAGGTGACAGGACTCAGCA-3′, and reverse, 5′-GGTCTCTGGATCCAACTCCA-3′. α_X_ forward, 5′-CTGGATAGCCTTTCTTCTGCTG-3′, and reverse, 5′-GCACACTGTGTCCGAACTCA-3′. GAPDH or 5S rRNA were used as an internal control (Ambion/Life Technologies, Grand Island, NY, United States).

### Statistical analysis

Statistical analyses were performed using Student's *t*-test or Student's paired *t*-tests where indicated in the text using SigmaPlot 13. A value of *p* < 0.05 was considered significant.

## Results

### Strong adhesion of classically-activated (M1) macrophages is converted in weak migration in contrast to well-migrated, but low-adherent alternatively-activated (M2) macrophages

**Figure 1:**
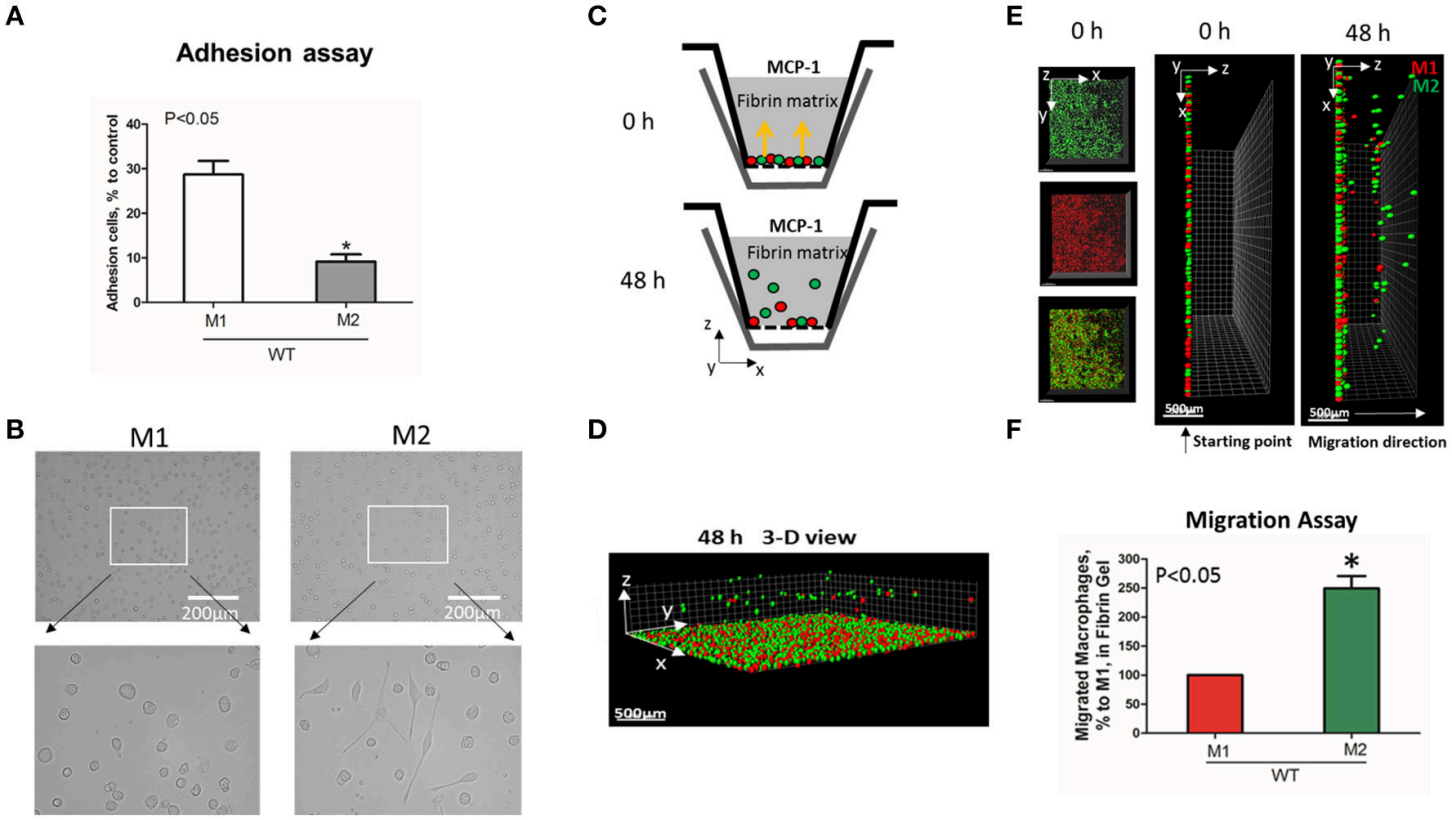
M1-activated macrophages demonstrate much stronger adhesive properties but weaker migration in comparison to M2-activated macrophages. **(A)** Adhesion assay of WT M1 and M2-activated macrophages to Fg. 96-well plate was coated with 4 μg/ml Fg for 3 h at 37°C. Fluorescently labeled M1 and M2 macrophages were added to the wells and cell adhesion was determined after 30 min in a fluorescence plate reader. Data are presented as mean ± SEM. *P < 0.05. **(B)** Morphologies of M1 (Left panel) and M2 (right panel) activated macrophages, scale bar = 200 μm. **(C–F)** 3-D migration assay in Fibrin matrix using M1 and M2 activated macrophages labeled with PKH26 (Red) and PKH67 (Green) fluorescent dyes, respectively. C. Sketch diagram of the migrating cells in Boyden transwell chamber. Before migration (upper panel) and after 48 h migration (lower panel). **(D)** 3-D view of the migrating cells in Fibrin matrix after 48 h. **(E)** Labeled Cells were mixed in equal amounts and verified by scanning samples with confocal microscope before the initiation of migration (**E**. left and middle panels). Migration of macrophages was stimulated by 30 nM MCP-1 added to the top of the gel. After 48 h, migrating cells were detected by a Leica Confocal microscope (**E**. right panel). **(F)** The results were analyzed by IMARIS 8.0 software and statistical analyses were performed using Student's paired *t*-tests (*n* = 4 per group). Scale bar = 500 μm. Data are presented as mean ± SEM. *P < 0.05.

**Figure 2:**
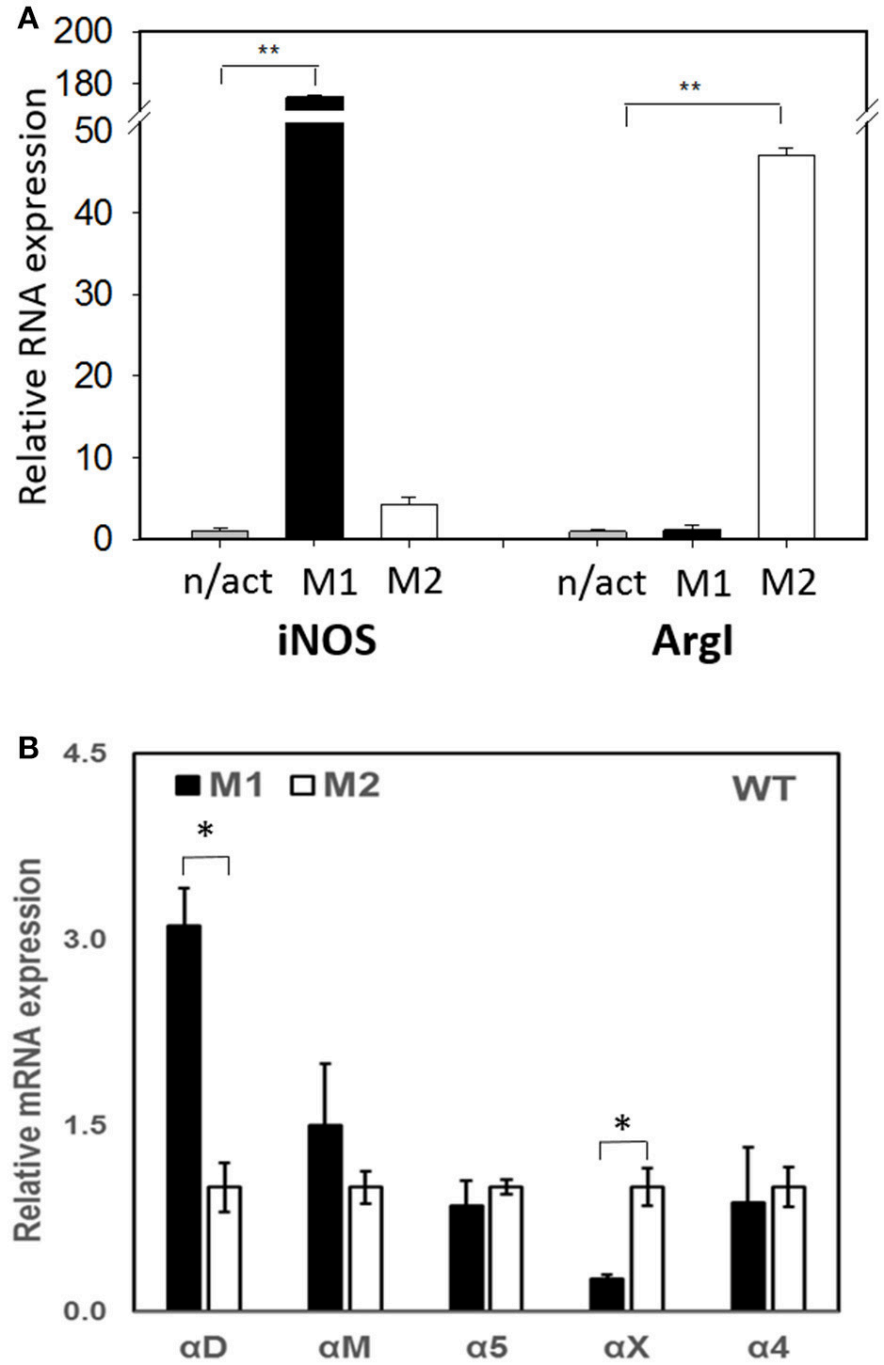
**(A)** The expression of M1 (iNOS) and M2 (Arg I) markers on IFN-γ (M1) and IL-4 (M2) stimulated macrophages using Real Time-PCR. Statistical analyses were performed using paired Student *t*-tests (*n* = 3 per group). Data are presented as mean ± SEM. **P < 0.01, compared to non-activated (n/act). **(B)** The expression of fibrin-binding integrins during M1 and M2 polarization. Open bars—non-activated; black bars M1-polarized, gray bars M2-polarized macrophages. Statistical analyses were performed using Student's paired *t*-tests (non-activated to activated) (*n* = 3 per group). Data are presented as mean ± SEM. *P < 0.05.

To evaluate the adhesive and migratory properties of M1 and M2 macrophages, we stimulated thioglycollate-induced peritoneal macrophages with IFNγ (M1-activated) or IL-4 (M2-activated) and evaluated the adhesion of these cells to fibrinogen and their migration in 3D fibrin matrix. The adhesion assay revealed a much stronger attachment of M1 macrophages (28.68 ± 5.33%) when compared to M2 macrophages (9.12 ± 2.79%) (Figure 1A). Moreover, M1 and M2 adherent cells possess different morphologies. While M1 macrophages have a rounded, flat, pancake-like shape after adhesion assay, M2 macrophages were elongated, and less spread out (Figure 1B). The development of M1 and M2 phenotypes were verified by upregulation of iNOS and ArgI, respectively (Figure 2A).

We tested how different adhesive properties affect macrophage cell migration (Figures 1C–F). Fluorescently labeled M1 (red, PKH26) and M2 (green, PKH67) macrophages were mixed in an equal number (Supplementary Figure 1A) and placed on a 3D fibrin gel where cell migration was stimulated via a MCP-1 gradient (Figures 1C,E). After 48 h, we detected a robust migration of M2 macrophages, which markedly exceeded the locomotion of M1 macrophages (Figures 1D–F). It has been shown previously that M1 and M2 macrophages demonstrate a similar chemotaxis to MCP-1 in 2D transwell assay (no ligand coated on membrane) (27). These data proved that the different migration of M1 and M2 macrophages in our 3D chemotaxis/haptokinesis assay does not regulated by different expression of CCR2 (chemotaxis), but by distinct adhesion-mediated migration (haptokinesis). To additionally verify it, the migration was repeated using a gradient of N-Formylmethionine-leucyl-phenylalanine (FMLP) and revealed similar results (Supplementary Figure 1B), therefore the adhesive receptors are potential cause of different migratory properties of M1 and M2.

## The levels of integrin expression determine the effects on macrophage migration

Recently, we demonstrated that integrin α_D_ is upregulated on M1-polarized macrophages but does not change on M2-polarized macrophages (23). We evaluated the potential changes in the expression of other fibrin-binding macrophage adhesive receptors during M1 and M2 polarization (Figure 2A). The RT-PCR results demonstrated that α_D_ is the only adhesive receptor that upregulates during M1 macrophage activation to compare with M2 subset (Figure 2B). We also detected the increased expression of integrin α_X_ on M2 macrophages; however, the total expression of α_X_ on macrophages is very low (20), which quashes its potential effect on macrophage migration. Therefore, the upregulation of integrin α_D_ is the most significant modification that may affect the migratory properties of M1 and M2 macrophages.

**Figure 3:**
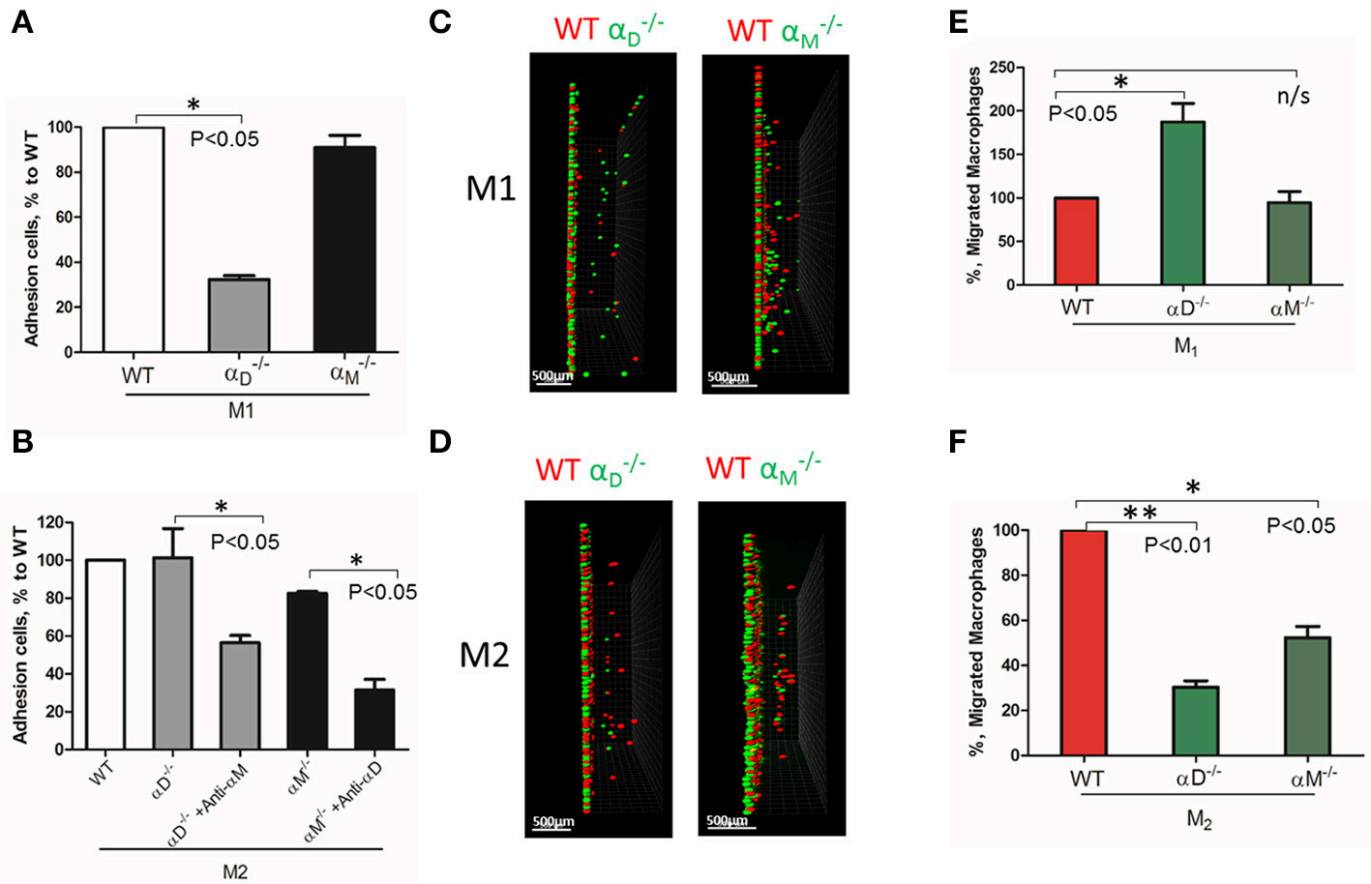
The level of integrin expression determines the effect on macrophage migration. **(A,B)** Adhesion assay to fibrinogen of WT, $\alpha_{\text{D}}^{-/-}$ and $\alpha_{\text{M}}^{-/-}$ macrophages activated to M1 **(A)** and M2 **(B)** phenotypes. Some samples in **(B)** were pre-incubated with anti-α_M_ and anti-α_D_ blocking antibodies before the adhesion assay. Data are presented as mean ± SEM. *P < 0.05. **(C,D)** Migration assay of α_D_- and α_M_-deficiency M1 **(C)** and M2 **(D)** macrophages in 3D fibrin matrix. After 48 h, migrating cells were detected by a Leica Confocal microscope and the results were analyzed by IMARIS 8.0 software, scale bar = 500 μm. **(E,F)** Statistical analyses were performed using Student's paired *t*-test (*n* = 4 per group). Data are presented as mean ± SEM. *P < 0.05, **P < 0.01.

Based on these data, further analysis was focused on integrin α_D_ and related integrin α_M_, that possess similar ligand binding properties, but distinct surface expressions. The contributions of integrin α_D_ and α_M_ to M1 and M2 migration were evaluated using α_D_- and α_M_-deficient macrophages. α_D_ deficiency reduced the adhesion of M1 macrophages to fibrinogen (Figure 3A), but significantly increased cell migration (Figures 3C, left panel; 3E). In contrast, integrin α_M_ deficiency has very limited effect on adhesion, due to its moderate expression on M1 macrophages (23) (Figure 2B), and did not demonstrate a significant effect on cell locomotion (Figures 3C,**E**). Both integrins, α_D_ and α_M_, have moderate expression on M2 macrophages (23) (Figure 2B). The adhesion of M2 macrophages depends on both integrins, which is demonstrated in the presence of antibodies and integrin-deficient cells (Figure 3B). In parallel assays, the reduced migration of α_M_- and α_D_-deficient macrophages verified that both integrins help to support the mesenchymal migration of M2 macrophages (Figures 3D,F).

The deficiency of α_D_ or α_M_ may also modify the expression of other fibrin-binding integrins that can affect cell migration. To test this possibility, we evaluated the expression of α_4_, α_5_, α_X_, and α_M_ on $\alpha_{\text{D}}^{-/-}$, as well as α_D_ on $\alpha_{\text{M}}^{-/-}$ macrophages activated to M1 and M2 phenotypes using RT-PCR. We did not detect any marked changes, except for the reduced expression of α_5_ and α_X_ on α_D_-deficient M1 macrophages (Supplementary Figure 2). Clearly, these changes cannot significantly modify migration.

## α_D_-mediated adhesion is critical for the retention of M1 macrophages

**Figure 4:**
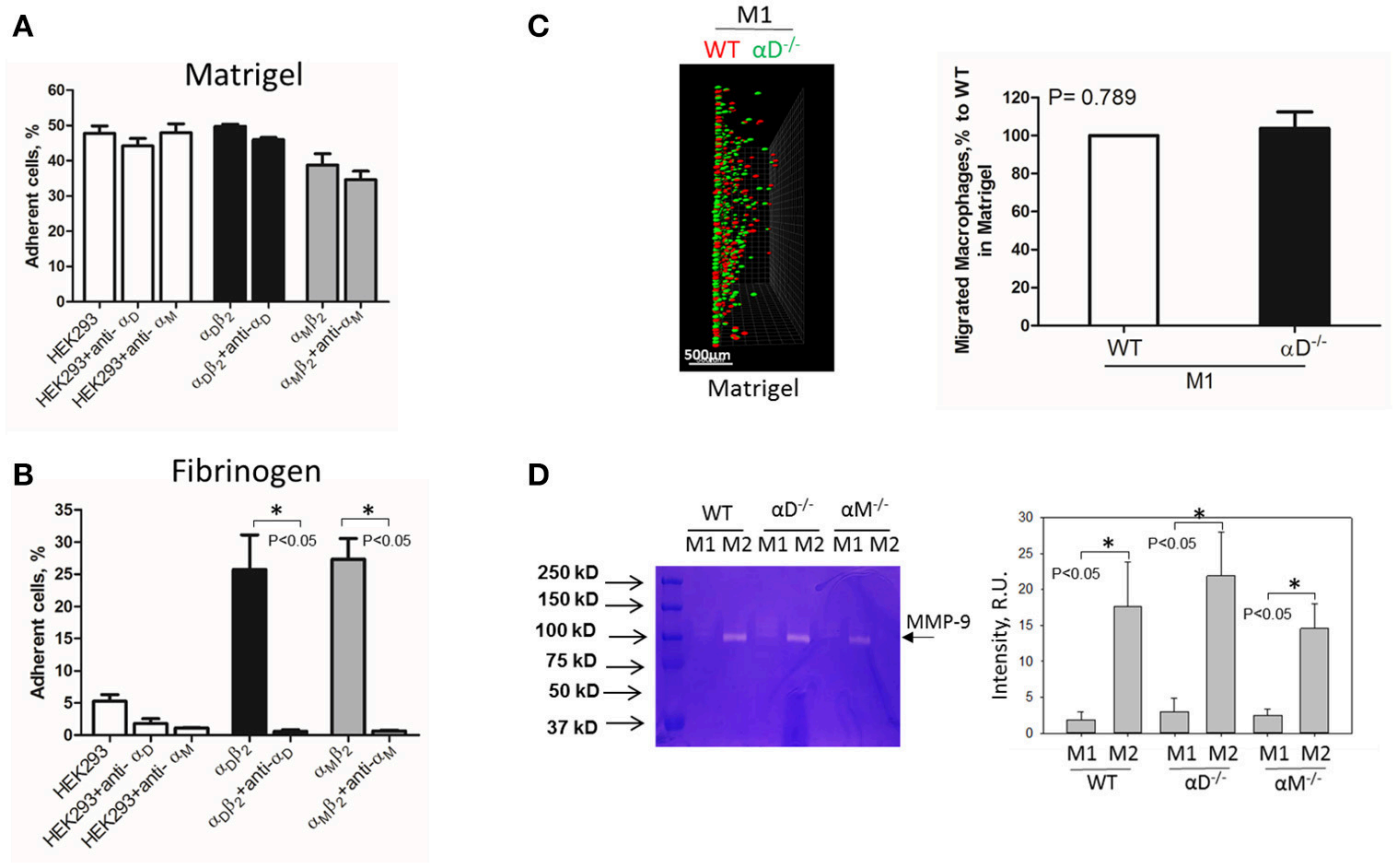
Matrigel does not support integrin α_D_-mediated adhesion and retention of M1 macrophages. **(A,B)** Adhesion of α_D_β_2_- and α_M_β_2_-transfected and mock-transfected HEK293 cells to Matrigel **(A)** and fibrinogen **(B)**. The adhesion was performed as described above. Data are presented as mean ± SEM. *P < 0.05. **(C)** 3-D migration assay of WT and α_D_-deficient M1 macrophages in Matrigel. Migration was stimulated by 30 nM MCP-1 added to the top of the gel. After 48 h, migrating cells were detected by a Leica Confocal microscope (Leica-TCS SP8) **(C**, left panel**)**. Scale bar = 500 μm. The results were analyzed by IMARIS 8.0 software. **(C**, right panel**)**. **(D)** Evaluation of MMPs in culture media after macrophage adhesion. WT, $\alpha_{\text{D}}^{-/-}$ and $\alpha_{\text{M}}^{-/-}$ M1- and M2-activated macrophages were plated on fibrinogen. Media was collected after overnight incubation and analyzed by gelatin-zymography (**D**, right panel). The intensity of gelatin degradation was evaluated by Fuji software (**D**, left panel). Statistical analyses were performed using Student's paired *t*-tests (*n* = 4 per group). Data are presented as mean ± SEM. *P < 0.05.

Inflamed extracellular matrix contains different β_2_ ligands, including fibronectin, vitronectin, thrombospondin, fibrinogen and others. Moreover, we recently showed that oxidative stress during inflammation may form ECM protein modifications with carboxyethylpyrole, which is also a ligand for β_2_ integrins (25). To verify the role of α_D_-mediated adhesion on cell migration, we performed macrophage migration in Matrigel, the model of basement membrane matrix, which consists of laminin, collagen IV and proteoglycans. Notably, these proteins are not ligands for integrin α_D_β_2_ or α_M_β_2_. To confirm this, we tested the adhesion of α_D_β_2_- and α_M_β_2_-transfected HEK293 cells to a plate coated with Matrigel (Figure 4A). Both cell lines demonstrated strong adhesion to Matrigel, but this adhesion was independent of α_D_ and α_M_, since anti-α_D_ and anti-α_M_ antibodies did not inhibit this binding. In contrast, the adhesion of α_M_β_2_ and α_D_β_2_-transfected cells to fibrinogen was significantly inhibited by these antibodies (21, 28) (Figure 4B). Apparently, the adhesion to Matrigel is mediated by integrins α_1_β_1_ and α_2_β_1_, which are receptors for laminin and collagen, and are expressed endogenously on HEK293 cells (29–31). To verify this hypothesis, we evaluated the adhesion of MOCK-transfected HEK293 cells to Matrigel and fibrinogen. These cells did not support the adhesion to fibrinogen, but demonstrated the same level of adhesion to Matrigel as α_D_β_2_ and α_M_β_2_ transfected cells (Figures 4A,B). Therefore, cells do not use α_D_β_2_ for the adhesion to Matrigel. Accordingly, we detected a similar level of wild type and α_D_-deficient M1 macrophage migration through Matrigel, which is distinct to our data in α_D_-dependent fibrin matrix. Therefore, this result is in agreement with our hypothesis regarding the critical role of α_D_-mediated adhesion for macrophage retention during 3D migration (Figure 4C).

However, one of the mechanisms that affects mesenchymal migration is the secretion of MMPs that degrade Matrigel. To test the potential effect of α_M_ or α_D_ deficiency on MMPs secretion, M1 and M2 macrophages were incubated in 48-well plates for 24 h and the media was tested using gelatin zymography as we described previously (32) (Figure 4D). First, we found a much stronger secretion of MMPs (specifically MMP-9) in M2 macrophages in comparison to M1 macrophages. Second, we did not detect any significant effect of α_D_- or α_M_-knockout on MMPs secretion, particularly in regard to M1-polarized macrophages.

**Figure 5:**
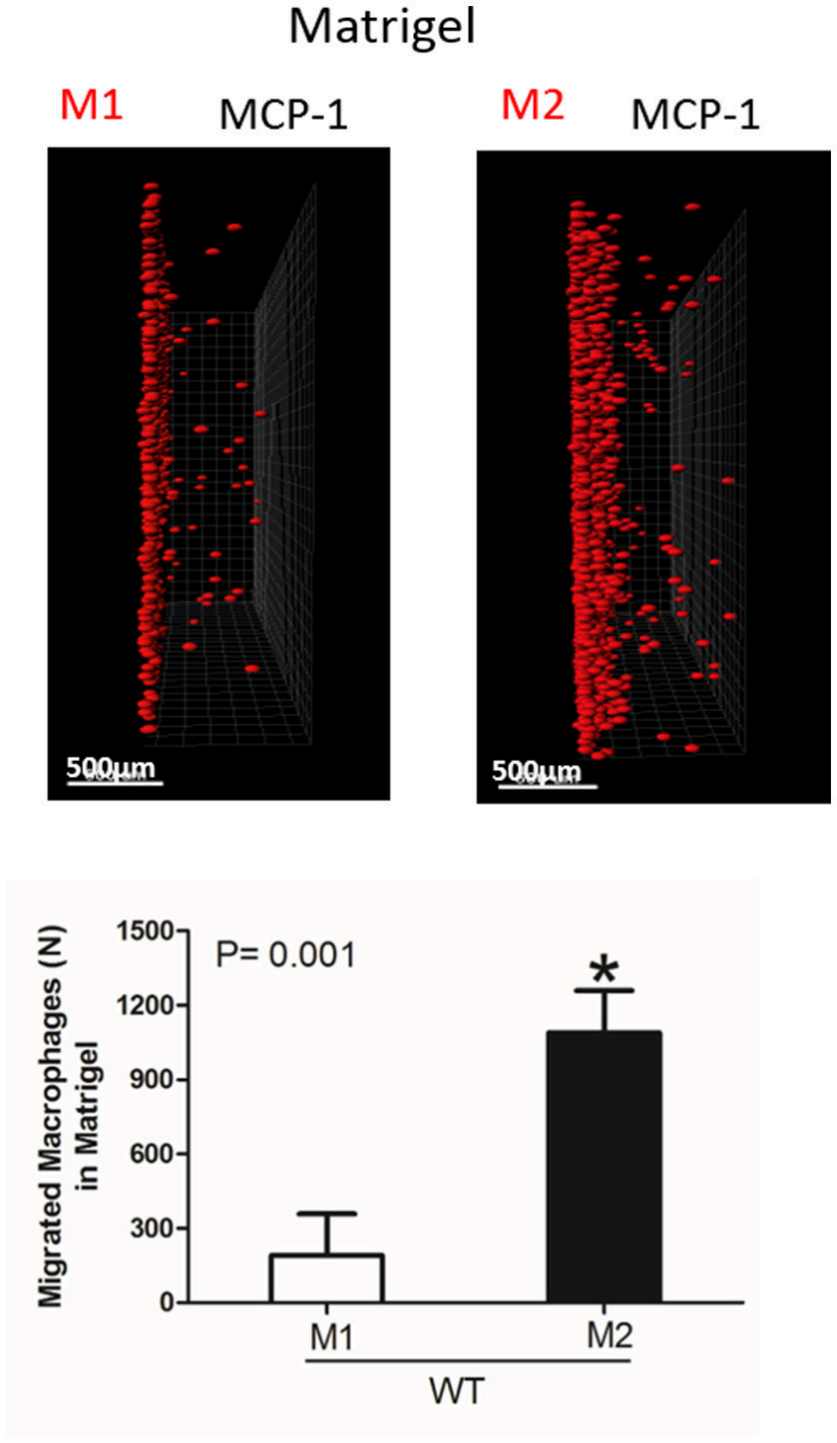
Migration of M1 and M2-activated macrophages in Matrigel. After 48 h, migrating cells were detected by a Leica Confocal microscope and the results were analyzed by IMARIS 8.0 software, scale bar= 500 μm. Statistical analyses were performed using Student's paired *t*-tests (*n* = 4 per group). Data are presented as mean ± SEM. *P < 0.05.

Interestingly, the robust secretion of collagen-specific MMP-9 by M2 macrophages can be responsible for the strong migration of these cells in Matrigel. The migration of M1 and M2 macrophages was performed in separate gels to avoid the effect of M2-released MMP-9 on the migration of M1 macrophages (Figure 5). In contrast, similar secretion of MMPs in WT and α_D_-deficient M1 macrophages allowed us to compare these two cell types in one sample. Therefore, the similar migration of WT and α_D_ macrophages in Matrigel was not regulated by a different level of MMPs secretion, but by the lack of α_D_-mediated adhesion.

## A high expression of α_M_ on resident macrophages reduces their amoeboid migration

**Figure 6:**
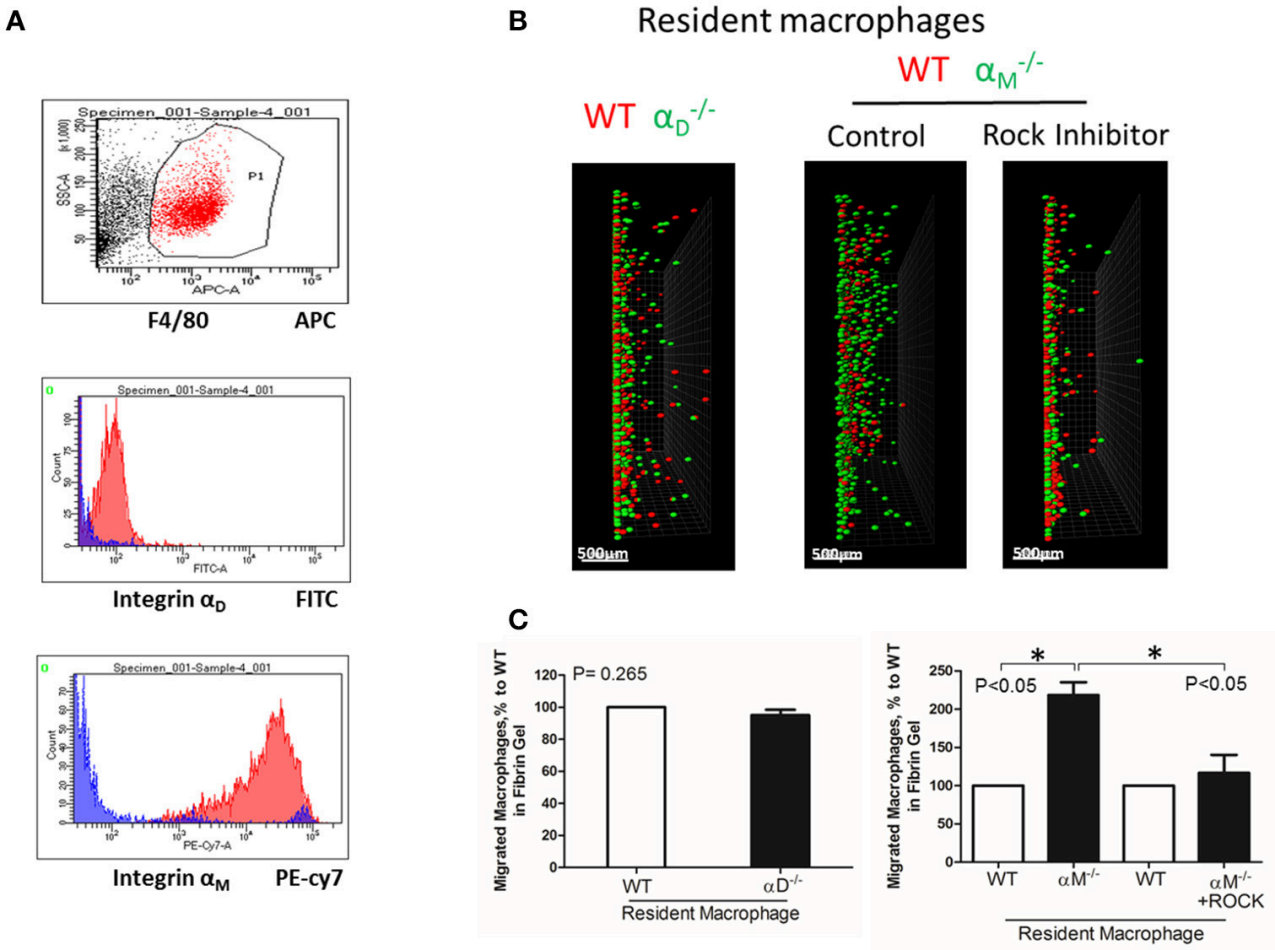
A high expression of α_M_ on resident macrophages reduces their amoeboid migration. **(A)** The expression of integrin α_D_ and α_M_ on resident macrophages was detected with anti-α_D_ and anti-α_M_ antibodies, respectively, and tested by flow cytometry analysis. **(B)** Migration of peritoneal resident macrophages in 3-D fibrin matrix. Migrating resident macrophages from WT and $\alpha_{\text{D}}^{-/-}$ mice are shown in the left panel. The middle and right panels represent the migrating resident macrophages from WT and $\alpha_{\text{M}}^{-/-}$ mice with or without Rock inhibitor (Y27632). Migrating cells were detected by a Leica Confocal microscope (Leica-TCS SP8). Scale bar = 500 μm. **(C)** The results were analyzed by IMARIS 8.0 software. Statistical analyses were performed using Student's paired *t*-tests (*n* = 4 per group). Data are presented as mean ± SEM. *P < 0.05.

To test the effect of high expression of other integrins on cell locomotion, we evaluated α_M_-dependent migration of resident macrophages. α_M_ has a very high expression on peritoneal resident macrophages (Figure 6A). A comparable analysis of 3D migration in fibrin matrix between WT and α_M_-deficient resident peritoneal macrophages revealed a strong improvement in the migration of the $\alpha_{\text{M}}^{-/-}$ subset (Figures 6B,C right panel). Notably, α_D_-deficiency, which has a very low expression on resident macrophages (Figure 6A), did not affect macrophage migration (Figures 6B,C left panel). These results demonstrated that α_M_ at high density on the cell surface can also prevent migration. It has been shown that resident macrophages apply the amoeboid migratory mode (24). Accordingly, the migration of WT and $\alpha_{\text{M}}^{-/-}$ in the presence of ROCK inhibitor, the inhibitor of amoeboid migration (33), resulted in a dramatic reduction in both the number of migrated cells and migratory distance (Figures 6B,C right panel). Therefore, macrophage adhesion-independent amoeboid migration can be reduced by integrin-mediated strong adhesion.

## *In vivo* migration of M1, M2, and resident macrophages confirmed the results of the 3D migration assays

**Figure 7:**
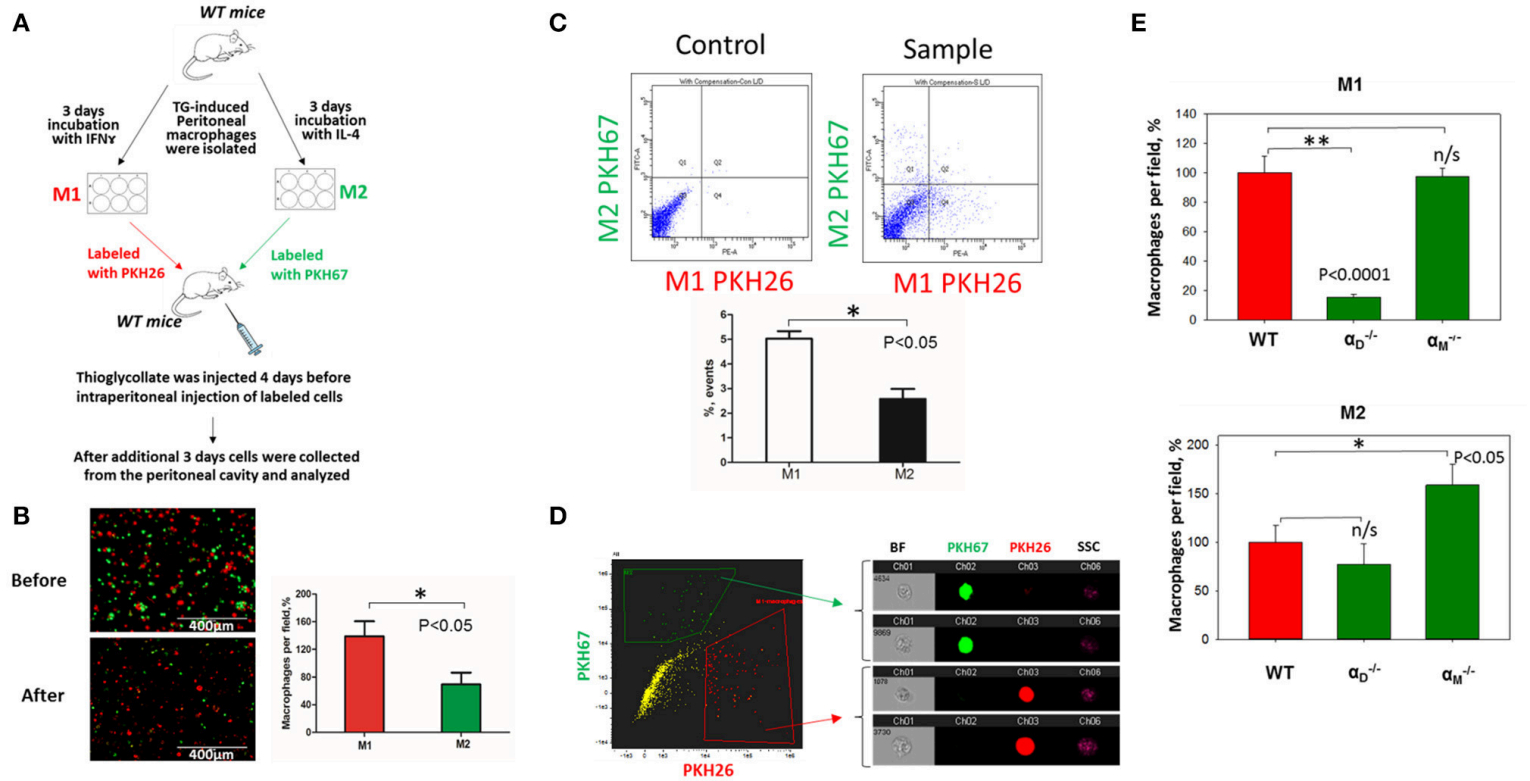
*In vivo* migration of M1 and M2 macrophages confirmed the results of the 3D migration assays. **(A)** The model of *in vivo* resolution of peritoneal inflammation. Peritoneal macrophages were isolated from WT mice at 3 days after injection of thioglycollate (TG) and placed on petri dish for 3 days incubation with 100 U/ml IFNγ or 2nM IL-4 to generate M1 and M2 activated macrophages, respectively. Collected M1 and M2 macrophages were labeled with PKH26 or PKH67 fluorescent dyes. Labeled M1 and M2 macrophages were mixed in a 1:1 ratio and further injected intraperitoneally into WT mice with 4 days predisposed TG-induced peritoneal inflammation. **(B)** The equal ratio of red and green macrophages before the injection was verified by sample cytospin preparation (**B**, upper panel). 3 days later, peritoneal macrophages were harvested, and the percentages of red and green fluorescent macrophages were assessed by cytospin (**B**, lower panel) and flow cytometry **(C,D)**. The quantification of the data was analyzed by using Image Analysis Software (EVOS, Thermo Fisher) at least 4 fields of view per sample (*n* = 4) (**B**, right panel). **(C)** Flow cytometry. Live isolated cells were selected with live/dead kit and analyzed using 488 and 567 channels (Fortessa X-20). The results were analyzed with Diva software and statistical analysis was performed using Student's *t*-test. Data are presented as mean ± SEM. *P < 0.05. **(D)** Imaging flow cytometer. The population of single, alive cells was analyzed on red and green channels and individual cells were evaluated in green and red positive areas (ImageStream Mark II, Amnis). Channel 1- Brightfield (BF). Channel 2- 488 wavelength (PKH67). Channel 3−566 wavelength (PKH26), channel 6—side scattering (SSC). **(E)** M1- and M2-activated macrophages in the peritoneal cavity during the resolution of peritoneal inflammation. The quantification of the data was analyzed by using Image Analysis Software (EVOS, Thermo Fisher) 4–6 fields of view per sample (*n* = 4). Data are presented as mean ± SEM. *P < 0.05.

To verify our *in vitro* results, we performed *in vivo* migration using the model of resolution of peritoneal inflammation as we have done previously (23). After the development of thioglycollate-induced peritoneal inflammation, macrophages migrate to, and accumulate within, the peritoneal cavity. The resolution of inflammation is started after 96 h and is characterized by the intensive emigration of macrophages from the peritoneal cavity to the lymphatics (34). We injected adoptively transferred M1 and M2 macrophages to assess their migratory properties in the *in vivo* environment (Figure 7A). *In vitro*-activated M1 and M2 macrophages were labeled with PKH26 and PKH67 fluorescent dyes, respectively. The recipient mice were first injected with thioglycollate and then, 96 h later, with an equal number of fluorescently labeled M1 and M2 macrophages. After an additional 72 h, the cells from the peritoneal cavity were collected and the number of M1 and M2 adoptively transferred macrophages was evaluated. The cytospin of harvested samples demonstrated the preferential accumulation of M1 macrophages (red fluorescence) in the peritoneal cavity (Figure 7B and Supplementary Figure 3), which corresponds to our *in vitro* migration assays (Figures 1D–F). Our FACS data confirmed these results, since mostly M1 macrophages reside in the peritoneal cavity, while M2 macrophages emigrate during resolution (5.02 ± 0.31% vs. 2.57 ± 0.41%) (Figure 7C). The Amnis imaging flow cytometry verified the size and morphology of fluorescently labeled macrophages in the peritoneal cavity (Figure 7D).

According to our *in vitro* results and previous data (23) we demonstrated that α_D_-deficiency on an M1 background stimulated the emigration of macrophages from the peritoneal cavity, while α_M_-knockout had no effect (Figure 7E). In contrast, we detected an increased accumulation of α_M_-deficient M2 macrophages in the cavity, which demonstrates the supportive role of α_M_ in the migration of M2 macrophages and remained consistent with our *in vitro* results. Surprisingly, we did not detect the same effect for $\alpha_{\text{D}}^{-/-}$ macrophages. The difference between the migrations of WT and $\alpha_{\text{D}}^{-/-}$ M2 macrophages was not significant (Figure 7E, lower panel).

**Figure 8:**
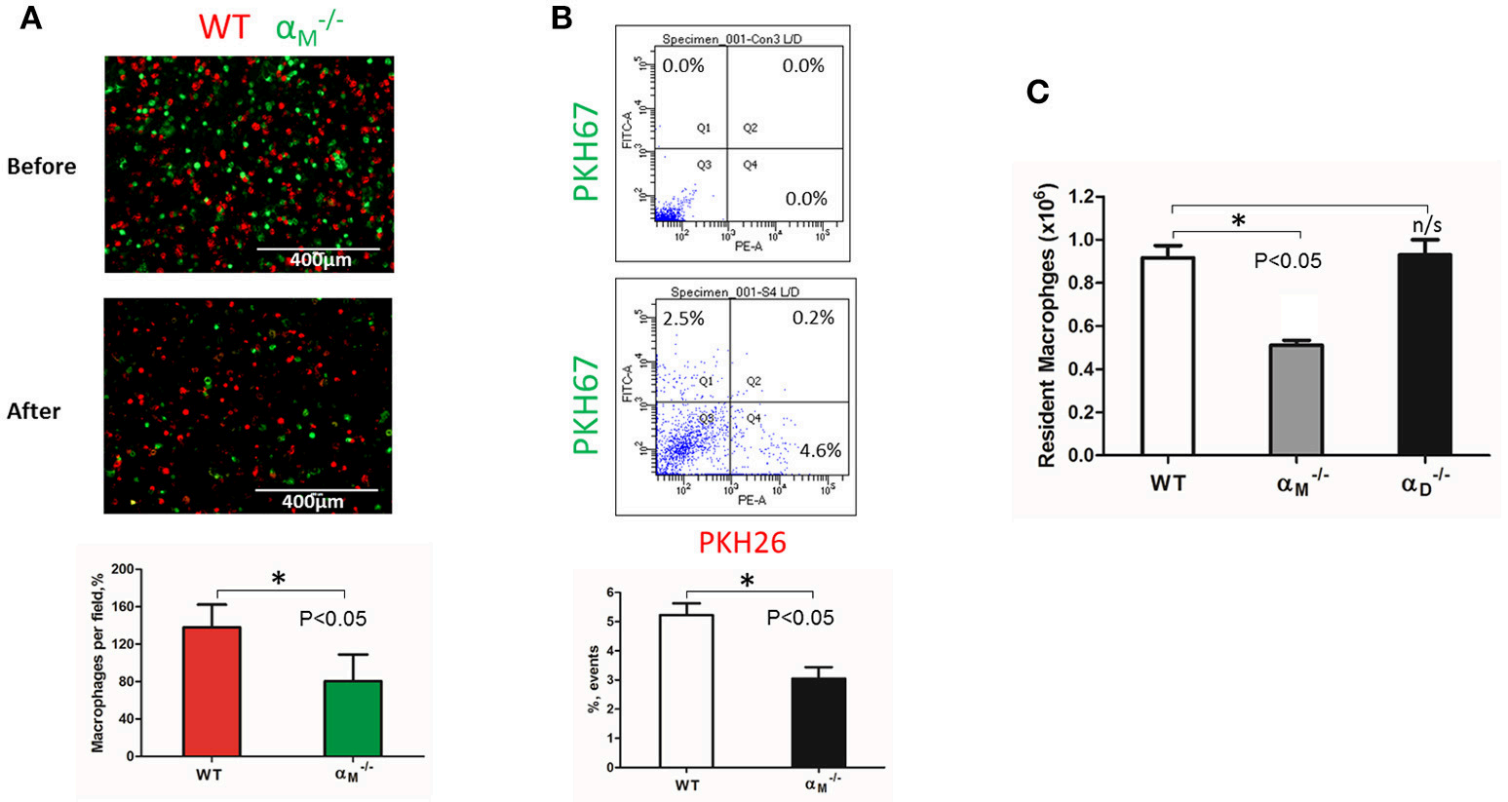
α_M_ deficiency improve efflux of resident macrophages. **(A)** Fluorescently-labeled resident peritoneal macrophages isolated from WT and $\alpha_{\text{M}}^{-/-}$ mice were mixed in equal numbers and confirmed by cytospin (**A**, upper panel). Labeled cells were injected introperitoneally into WT mice 4 days after TG-induced inflammation. After 3 days, the harvested peritoneal cells were cytospun (**A**, middle panel). The quantification of the data was analyzed using *t*-test at least 4 fields of view per sample (*n* = 4) by Image Analysis Software (EVOS, Thermo Fisher) (**A**, lower panel). Data are presented as mean ± SEM. *P < 0.05. **(B)** The harvested macrophages were also assessed by flow cytometry and the percentages of red (Q4) and green (Q1) fluorescent cells were assessed. Data are presented as mean ± SEM. *P < 0.05. **(C)** The amount of resident WT, $\alpha_{\text{M}}^{-/-}$ and $\alpha_{\text{D}}^{-/-}$ macrophages was evaluated by assessing the number and percentage of macrophages in non-inflamed peritoneal cavity of mice. Isolated peritoneal cells were counted and the number of WT, $\alpha_{\text{M}}^{-/-}$ and $\alpha_{\text{D}}^{-/-}$ resident macrophages were calculated based on the percentage of F4/80 positive population in flow cytometry analysis. Data are presented as mean ± SEM. *P < 0.05.

WT and $\alpha_{\text{M}}^{-/-}$ resident macrophages were isolated and tested using the same resolution of inflammation assay. After 72 h, we detected predominantly wild type cells in the peritoneal cavity, while α_M_-deficient macrophages emigrated (Figure 8A). This result was verified by flow cytometry. The number of red-fluorescent WT cells isolated from the peritoneal cavity significantly exceeded the number of green-fluorescent $\alpha_{\text{M}}^{-/-}$ cells (Q4 vs. Q1), (Figure 8B). Based on this result, we suggest that α_M_ serves for the supporting resident macrophage accumulation in the tissue, and α_M_-deficiency increases the efflux of resident macrophages.

To confirm this conclusion, we evaluated the number of macrophages in the non-inflamed peritoneal cavity of wild type and $\alpha_{\text{M}}^{-/-}$ mice. Isolated peritoneal cells were stained with F4/80 antibodies and analyzed by flow cytometry to detect the percentage of macrophages. We found that α_M_-deficiency resulted in a twofold reduction in the number of resident macrophages in the cavity (Figure 8C). In contrast, α_D_-deficiency on resident peritoneal macrophages did not affect macrophage number. These data are in agreement with our *in vitro* and *in vivo* migration assays.

## α_D_ deficiency reduces macrophage accumulation in adipose tissue and improves metabolic parameters

**Figure 9:**
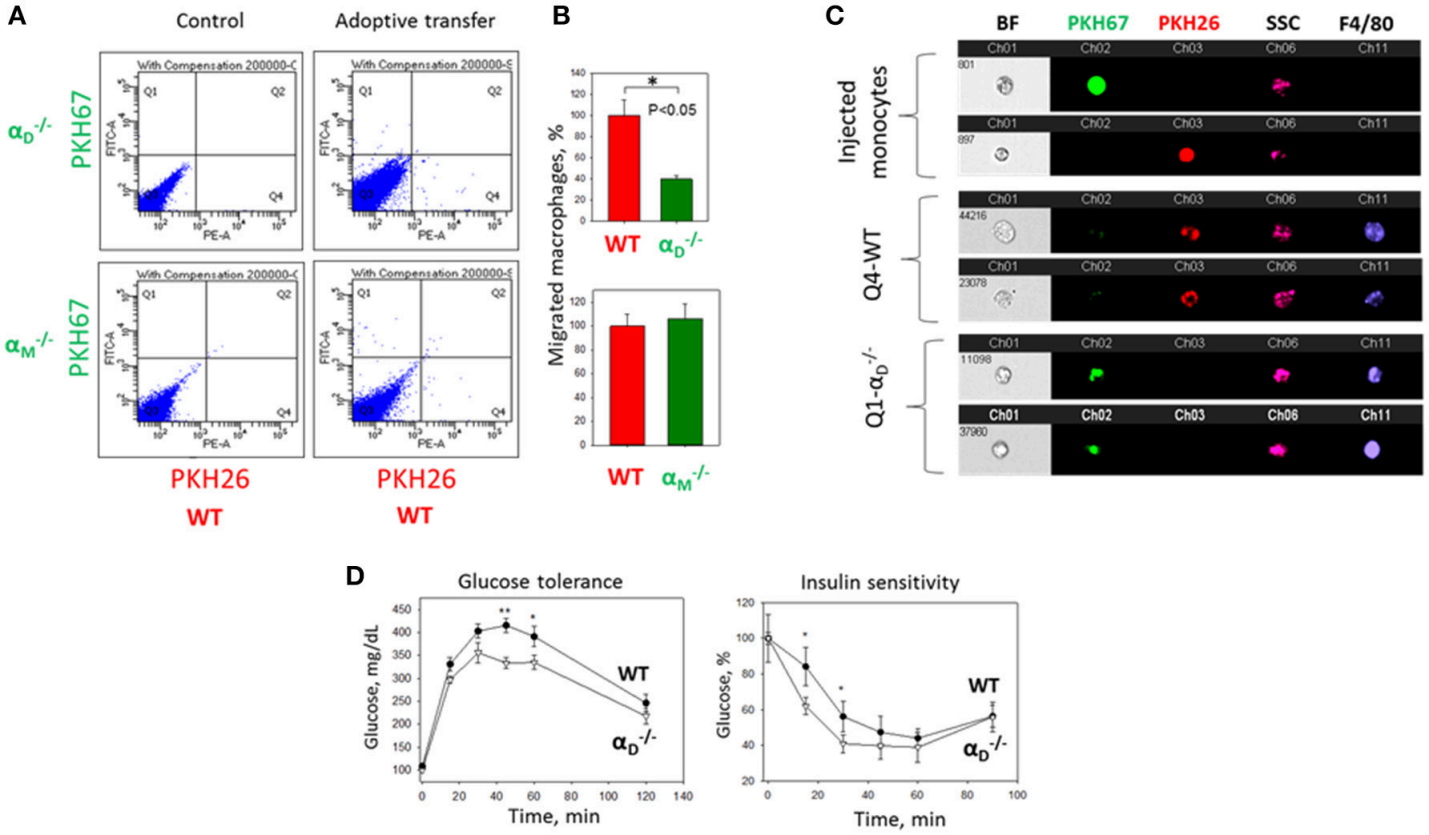
α_D_ deficiency reduces accumulation of monocyte-derived macrophages in adipose tissue and improves metabolic parameters during diet-induced diabetes. **(A)** WT and $\alpha_{\text{D}}^{-/-}$ (or $\alpha_{\text{M}}^{-/-}$) monocytes were isolated from bone marrow, labeled with red (WT) or green ($\alpha_{\text{D}}^{-/-}$) fluorescent dyes, respectively, mixed in an equal amount and injected into the tail vein of WT mice fed for 8 weeks with high fat diet (45% kcal/fat). After 3 days visceral adipose tissue was isolated, digested and analyzed using flow cytometry. **(B)** Statistical analyses were performed using Student's paired *t*-tests (*n* = 4 per group). Data are presented as mean ± SEM. *P < 0.05. **(C)** Imaging flow cytometry. Upper panel represents the injected monocytes, isolated from WT and $\alpha_{\text{D}}^{-/-}$ (or $\alpha_{\text{M}}^{-/-}$) mice, labeled with red and green fluorescent dyes, respectively. Middle (Q4) and lower(Q1) panels represent the labeled cells in digested adipose tissue. Channel 11- F4/80 represents macrophage staining. **(D)** WT mice (black circles) and α_D_-knockout mice (white triangles) were fed with high fat diet for 16 weeks and glucose intolerance (left panel) and insulin resistance (right panel) were evaluated. *N* = 6 for $\alpha_{\text{D}}^{-/-}$ and *n* = 9 for WT per group. A statistical analysis was performed using Student's *t*-test. Data are presented as mean ± SEM. *P < 0.05; **P < 0.01, compared to $\alpha_{\text{D}}^{-/-}$ group.

To further confirm the contribution of α_D_β_2_ to macrophage retention in the site of chronic inflammation, we used the model of diet-induced diabetes. The accumulation of pro-inflammatory (M1-like macrophages) in the inflamed adipose tissue is a hallmark of the inflammatory component of diabetes (26). It has been shown that α_D_ is upregulated in the adipose tissue during diet-induced obesity (35), which concurs with the upregulation of α_D_ on M1-activated macrophages *in vitro* and in atherosclerotic lesions (23). We also detected a strong expression of α_D_β_2_ on adipose tissue macrophages of C57BL6 mice after 8 weeks of a high fat diet (45 kcal% fat) (Supplementary Figures 4A,B). To assess the role of α_D_β_2_ and α_M_β_2_ in macrophage migration during chronic inflammation, monocytes isolated from WT and $\alpha_{\text{D}}^{-/-}$ (or $\alpha_{\text{M}}^{-/-}$) mice were labeled with red (PKH26) or green (PKH67) dyes, respectively, mixed in equal number and injected intravenously into mice on a high fat diet (Supplementary Figure 4C). The accumulation of adoptively transferred WT and integrin-deficient macrophages in the adipose tissue of these mice was evaluated after 3 days. The isolated adipose tissue was digested and analyzed by multi-color FACS. We detected a 3-fold decrease in the number of α_D_-deficient macrophages (in comparison to WT) in the visceral adipose tissue (Figures 9A,B). The result was verified by Imaging flow cytometry that confirmed the presence of labeled cells in the digested adipose tissue (Figure 9C). More importantly, it also demonstrates the maturation of labeled macrophages, since migrated cells expressed macrophage receptor F4/80 (Figure 9C, Lower panels), while injected monocytes lack this expression (Figure 9C, Upper panel). Interestingly, the deficiency of integrin α_M_, which did not significantly upregulate on M1 macrophages (23) (Figure 2B) had no effect on macrophage accumulation in adipose tissue (Figure 9A, Lower panel). Our previous data demonstrate that α_D_ deficiency does not affect monocyte recruitment from circulation during inflammation (23). Therefore, these results are in agreement with our *in vitro* and *in vivo* experiments and with recently published data that α_M_ deficiency does not affect the accumulation of macrophages during diet-induced obesity (36, 37).

The assessment of metabolic parameters of α_D_-knockout and WT mice after 16 weeks on a high fat diet confirm the physiological significance of our results by showing that a reduced number of macrophages in the adipose tissue of $\alpha_{\text{D}}^{-/-}$ improved glucose tolerance and insulin sensitivity (Figure 9D). On the other hand, the recently published data did not reveal a change in glucose tolerance test of α_M_-deficient mice in comparison to WT control after 20 weeks of high-fat diet, but detected decreased insulin sensitivity in skeletal muscle and liver (37).

Taken together, these results provide the link between integrin expression and potential pathophysiological functions. Apparently, the same integrin can support or inhibit 3D migration in tissue depending on the macrophage subset and the level of integrin expression on the cell surface.

## Discussion

The accumulation of macrophages at the site of inflammation is a complex physiological process that is critical for the development and resolution of inflammation. Macrophage apoptosis, proliferation and chemokine stimulation are important components of this mechanism, but the adhesive receptors that regulate the macrophage accumulation via cell migration and cell retention are the critical factors that generate the final outcome.

During the last decade, the role of adhesive receptors, particularly integrins, in the three-dimensional migration of immune cells in tissue has been questioned due to a new mechanism, the amoeboid mode of migration, being suggested (12, 38). However, recent data demonstrate that some immune cells, particularly macrophages, utilize adhesion-mediated mesenchymal migration in 3D matrices (13, 39). It has been shown that the migratory mode of macrophages depends on the environment and density of matrix (33). Previously, based on 2D models, it was suggested that cell migration is regulated by cell-substratum adhesiveness, which depends on substrate concentration, adhesive receptor density and affinity (15). This theory postulates that an intermediate level of adhesiveness (or intermediate expression of the adhesive receptors) is optimal for cell migration, while very low adhesiveness does not support cell locomotion and very high adhesiveness inhibits migration due to the prevention of the detachment of adhered cells. However, this theory was not evaluated during 3D migration in the tissue, which has more complex regulatory mechanisms and much stronger physiological implications. In this project, we tested integrins α_M_β_2_ and α_D_β_2_ as physiologically relevant models for studying the role of adhesive receptors during the migration of different subsets of macrophages. We discussed resident peritoneal macrophages and two subsets of monocyte-derived activated macrophages—classically activated (called M1), which can be generated by IFNγ/LPS or TNFα stimulation; and alternatively activated, which are produced by stimulation with IL-4 and/or IL-13 (called M2a) (7). For simplicity, we are calling the latter group M2. We realize that M1 and M2 activated macrophages are simplified models; and macrophages in the atherosclerotic lesion and adipose tissue may represent “mixed phenotypes.” However, these two subsets characterize the most variable difference in macrophage functional properties, and therefore, are an appropriate model for analyzing β_2_ integrin expression and functions in different macrophage subsets.

Our experimental approach is based on several observations. First, α_D_ and α_M_ share similar ligands (21, 22); second, these two integrins form a complex with the same β_2_ subunit, thus leading to similar integrin-mediated outside-in signaling during the interaction with the ligand; and third, the expressions of α_D_ and α_M_ are distinct on M1-polarized, M2-polarized and resident macrophages. We demonstrated that α_D_ is upregulated on M1 macrophages, while the expression of α_M_ is moderate (Figure 2) and (23). In contrast to these observations, the resident macrophages express a low level of α_D_, but have a high density of α_M_ (Figure 6). At the same time, the expressions of both α_D_ and α_M_ integrins on M2 macrophages are intermediate (Figure 2).

Using these three subsets of macrophages, we found that 1) M2 macrophages possess much stronger migratory ability within 3D matrix in comparison with M1. 2) Integrins α_D_β_2_ and α_M_β_2_ are important receptors that regulate cell migration. 3) Similar to the 2D migration, integrins can support mesenchymal 3D cell migration at the intermediate density and prevent mesenchymal and amoeboid cell migration at high levels of expression. 4) Even the adhesion-independent amoeboid mode can be negatively-regulated by a high expression of β_2_ integrins.

In this project, we show that strong adhesion via integrins is critical for cell retention that defines the different migratory properties of M1 and M2 macrophages. (Figures 3, 6). The analysis of α_M_, α_X_, α_D_, α_5_, and α_4_, integrins demonstrates that the upregulation of α_D_ on M1 macrophages is a major change in integrin expression during M1 activation. Therefore, α_D_β_2_-mediated adhesion is crucial for the prevention of M1 macrophage migration. In a parallel line of evidence, we found that the lack of α_D_-dependent substrate (exemplified in Matrigel) eliminates the effect of α_D_ on cell migration in this matrix (Figure 4). Importantly, α_D_-deficiency does not significantly change the expression of other macrophage integrins and the levels of MMP expression, which rules out the possibility for an indirect effect of α_D_ knockout on M1 macrophage migration.

Taken together, these results propose that the accumulation of M1 macrophages at the site of inflammation is mediated by strong adhesion which promotes cell retention and the progression of chronic inflammation. In agreement with that, α_D_-deficiency prevents the accumulation of adoptively transferred fluorescently-labeled macrophage accumulation in adipose tissue during diabetes. The reduced number of macrophages is associated with reduced inflammation and improved glucose tolerance and insulin sensitivity in α_D_-knockout mice. These data correspond to our previous results, that α_D_-deficiency reduced macrophage accumulation in atherosclerotic lesions and the development of atherosclerosis (23). Therefore, the upregulation of α_D_ on pro-inflammatory macrophages during diabetes (35) or atherosclerosis (23) demonstrates a similar outcome, which is manifested in the macrophage retention at the site of inflammation and disease development. Interestingly, α_M_ deficiency has pro-atherogenic effect on female and no effect on male mice (40). In agreement with this result, it has been recently shown that α_M_ deficiency elevates glucose level and decreased insulin sensitivity after 16 weeks on a high fat diet. Taken together, these data confirm the opposite role of α_D_β_2_ and α_M_β_2_ on pro-inflammatory M1 macrophages.

In contrast, the stronger migratory properties of M2 macrophages indicate that these cells more easily leave the tissue toward the lymphatics. The increased phagocytic properties of M2 macrophages, coupled with their high migratory abilities, confirm the major function of anti-inflammatory macrophages—phagocytosis followed by efflux from the tissue. α_D_ and α_M_ support the motility of M2 macrophages, and therefore promote the emigration of M2 macrophages from the inflamed tissue. Interestingly, the role of α_M_ in macrophage efflux during resolution was proposed previously (41).

The published data demonstrates that M2 macrophages may apply both locomotion modes, amoeboid and mesenchymal, which is supported by our observations regarding the α_M_ and partially α_D_-mediated mesenchymal migration of M2 macrophages (Figure 3). In contrast, resident macrophages use preferentially amoeboid motility. Using ROCK inhibitor, we confirmed the preferential amoeboid migration of resident macrophages, but also demonstrated that amoeboid migration can be increased after the knockout of α_M_ integrin, which has a high density on these cells (Figure 6). Therefore, these data propose an anchoring role for integrin α_M_β_2_ for resident macrophages in tissue. This mechanism may be important for the normal homeostasis and mobilization the initial immune defense, which is mediated by resident macrophages. We showed that α_M_-deficiency reduced macrophage numbers in the non-inflamed peritoneal cavity (Figure 6). Therefore, the different immune pathologies associated with α_M_-deficiency can be at least partially related to the impaired resident macrophage number. Most importantly, since integrins can block (or reduce) amoeboid migration, it suggests the potential role of integrins in the regulation (particularly, inhibition) of 3D migration of other immune cells that use only amoeboid movement (for example neutrophils or dendritic cells).

In summary, our study demonstrates the important contribution of α_D_β_2_ and α_M_β_2_ to the locomotion of distinct macrophage subsets and proposes a β_2_-integrin dependent mechanism of macrophage retention in the tissue and efflux during the resolution of inflammation.

## Author contributions

KC designed and performed the experiments, analyzed the data and edited the manuscript. CA performed the experiments and analyzed the data. NP analyzed the data and edited the manuscript. VY designed the research, performed the experiments, analyzed the data, and wrote the manuscript.

### Conflict of interest statement

The authors declare that the research was conducted in the absence of any commercial or financial relationships that could be construed as a potential conflict of interest.

## Abbreviations

ECM: Extracellular matrix
EDTA: Ethylenediaminetetraacetic acid
FACS: Fluorescence-activated cell sorting
FMLP: N-Formylmethionine-leucyl-phenylalanine
IFNγ: interferon-γ
IL-4: interleukin 4
MCP-1: Monocyte chemoattractant protein-1
ROCK: Rho-associated protein kinase
TG: thioglycollate
WT: wide type
2D: 2 dimensional
3D: 3 dimensional.
