# Supplementary figures and images for: Distinct Migratory Properties of M1, M2, and Resident Macrophages Are Regulated by αDβ2 and αMβ2 Integrin-Mediated Adhesion
Source: Front Immunol. 2018 Nov 15;9:2650. doi: 10.3389/fimmu.2018.02650 (PMC6262406; doi:10.3389/fimmu.2018.02650)

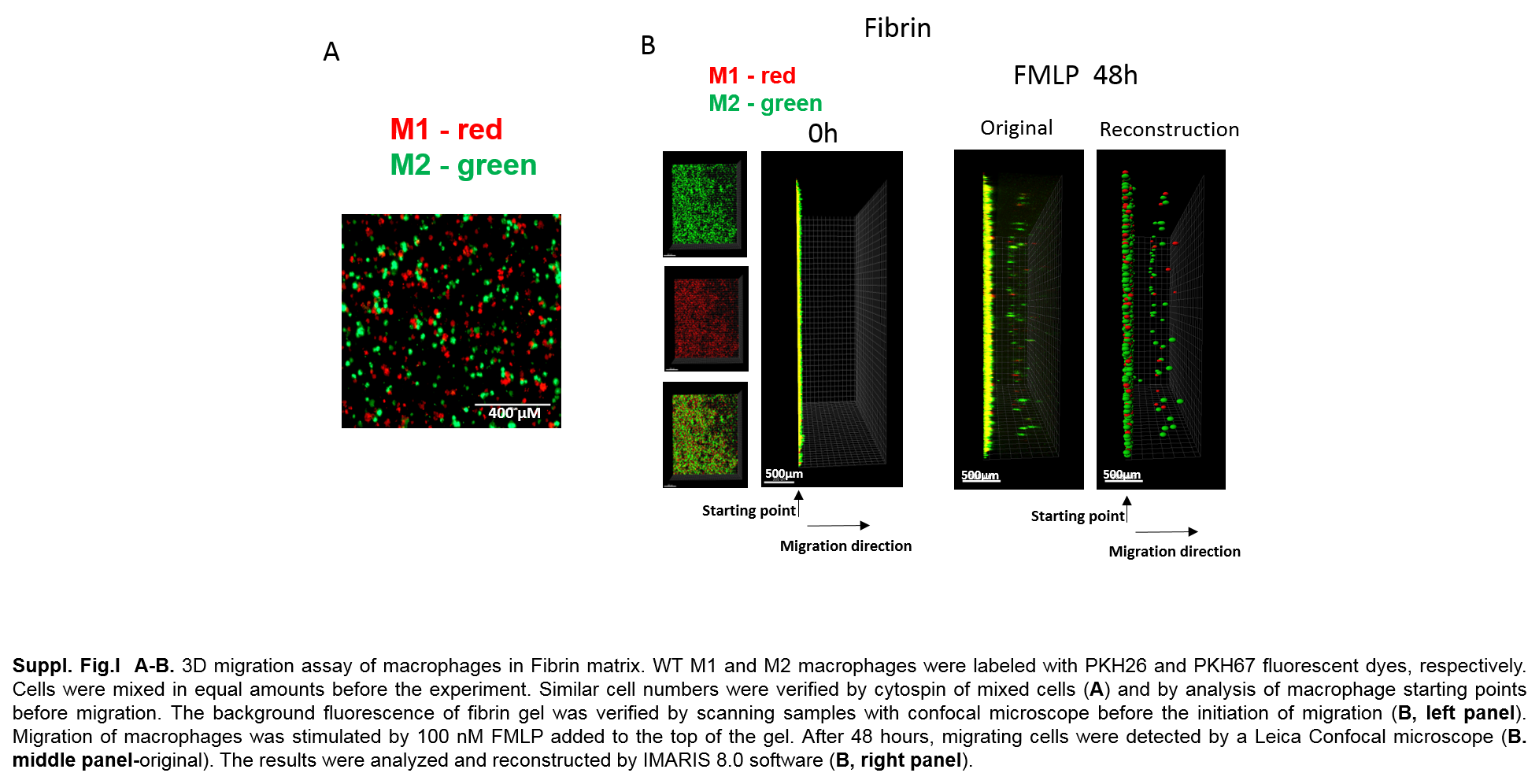

Supplement: Supplementary file 1 [file Image_1.TIF]

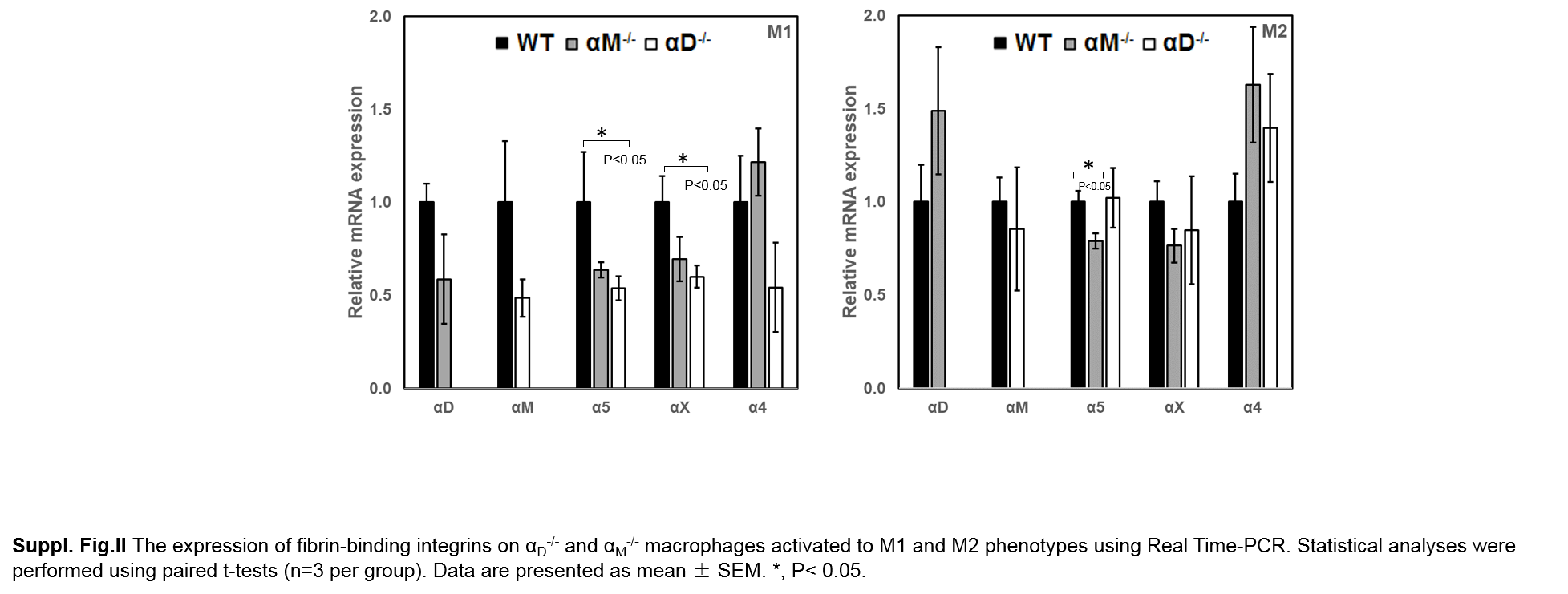

Supplement: Supplementary file 2 [file Image_2.TIF]

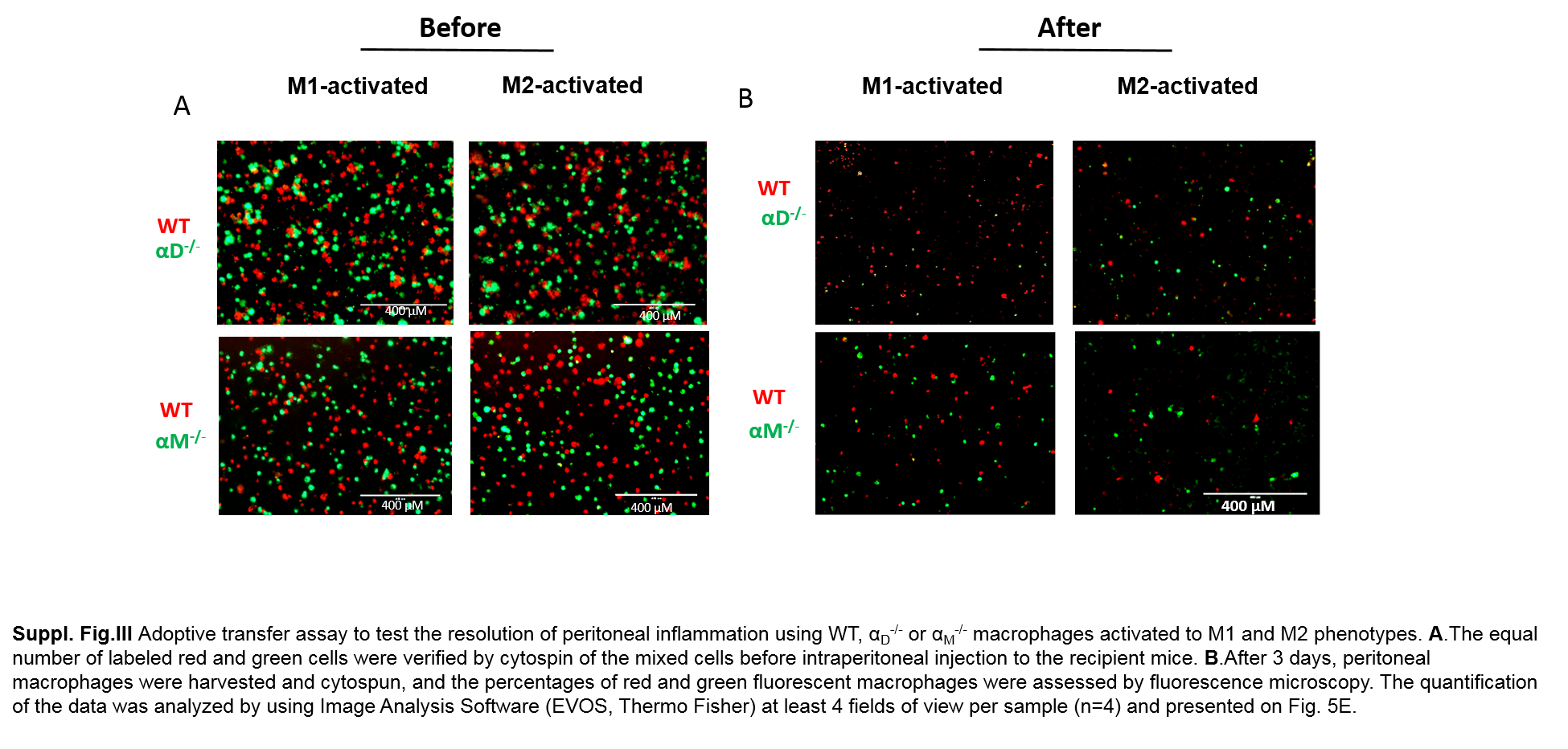

Supplement: Supplementary file 3 [file Image_3.TIF]

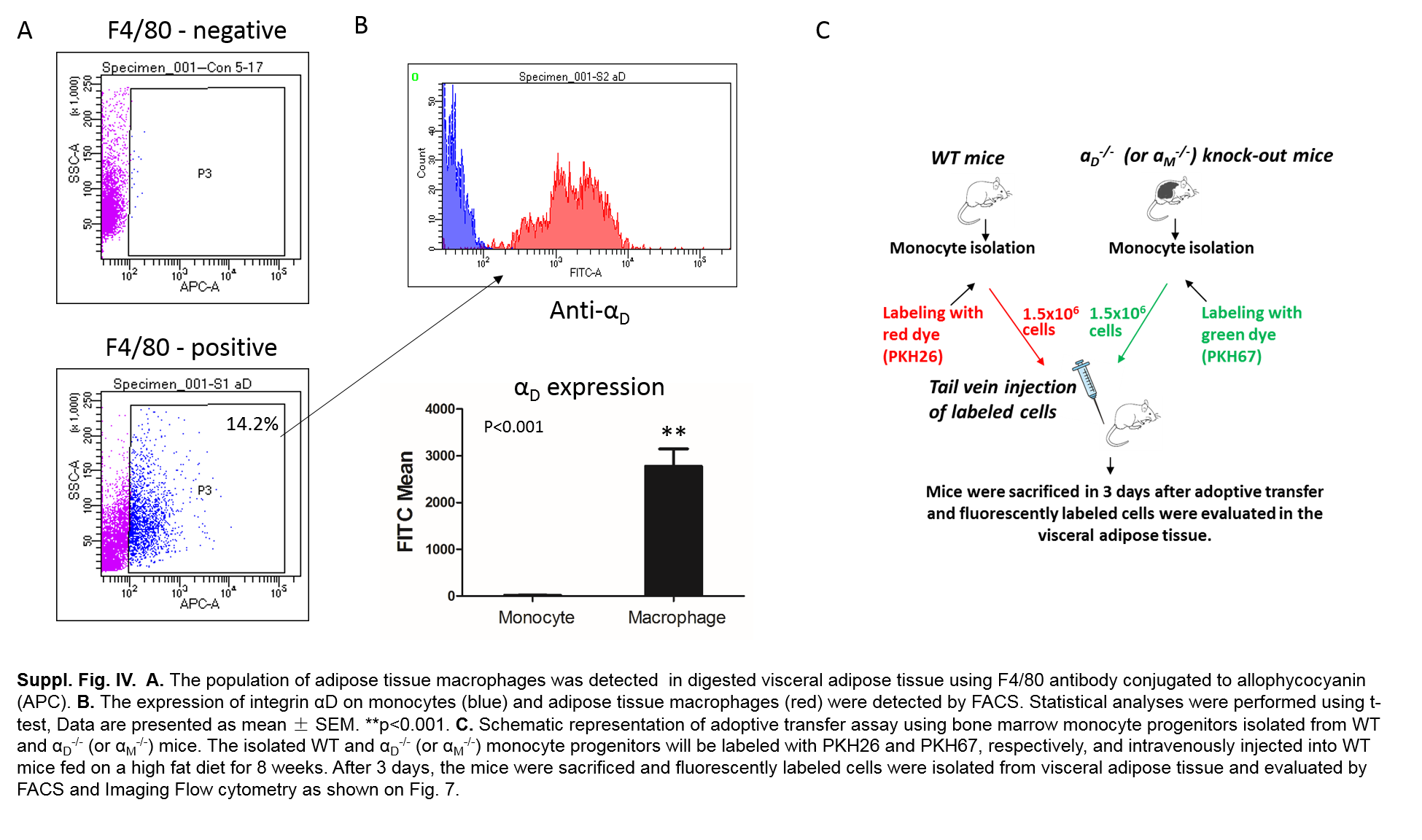

Supplement: Supplementary file 4 [file Image_4.TIF]
